# Efficacy and safety of combined immunotherapy and stereotactic radiosurgery in NSCLCBM patients and a novel prognostic nomogram: A real-world study

**DOI:** 10.3389/fonc.2023.1068592

**Published:** 2023-04-14

**Authors:** Shoaib Bashir, Lei Wen, Ping Zhang, Minting Ye, Yin Li, Weiping Hong, Junjie Zhen, Mingyao Lai, Hui Wang, Yanying Yang, Xingrui Chen, Rishun Luo, Guoxia Jia, Yao Guo, Linbo Cai, Meng Xu

**Affiliations:** ^1^ Oncology Department, First Affiliated Hospital of Jinan University, Guangzhou, China; ^2^ Oncology Department, Guangdong Sanjiu Brain Hospital, Guangzhou, China

**Keywords:** non-small cell lung cancer, brain metastases, stereotactic radiosurgery, immunotherapy, prognostic nomogram

## Abstract

**Objective:**

To explore the effectiveness of combined immunotherapy (IT) and stereotactic radiosurgery (SRS) and address the gap between evidence-based clinical practice and academic knowledge of optimal timing of IT relative to SRS. In addition, to meet the unmet need for an up-to-date prognostic assessment model in the era of IT.

**Methods:**

The data of 86 non-small cell lung cancer brain metastasis (NSCLCBM) patients treated with SRS to 268 brain metastases (BMs) were retrospectively extracted from our hospital database. The Kaplan–Meier analysis was employed for overall survival (OS) and a log-rank test for comparison between groups. Cox proportional hazards regression models were used to identify the significant prognostic factors. The prognostic nomogram was established utilizing the rms package of R software.

**Results:**

IT was found to be associated with improved OS (from BM diagnosis: HR 0.363, 95% CI 0.199 - 0.661, *P* < 0.001; from SRS: HR 0.472, 95% CI 0.260 - 0.857, *P* = 0.014). Individuals who received IT in combination with SRS had better OS than those who didn’t (from the day of BM diagnosis: 16.8 vs. 8.4 months, *P* = 0.006; from the day of SRS: 12 vs. 7 months, *P* = 0.037). Peri-SRS timing of IT administration was a significant prognostic factor for OS (from BM diagnosis: HR 0.132, 95% CI 0.034 - 0.517, *P* = 0.004; from SRS: HR 0.14, 95% CI 0.044 - 0.450, *P* = 0.001). Initiating IT after SRS led to superior OS than concurrent or before (from BM diagnosis: 26.5 vs. 14.1 vs. 7.1 months; from SRS: 21.4 vs. 9.9 vs. 4.1 months, respectively). Additionally, we build a nomogram incorporating IT, cumulative intracranial tumor volume (CITV), and recursive partitioning analysis (RPA), demonstrating a remarkable prognosis prediction performance for SRS-treated NSCLCBM patients.

**Conclusion:**

Peri-SRS IT is a promising approach in treating NSCLCBM, as improved OS was observed without significantly increasing adverse events. Receipt of IT post-SRS was associated with superior OS than those who received IT concurrently or before. Incorporating IT and CITV into the RPA index could augment its prognosis assessment value for SRS-treated NSCLCBM patients, predominantly in the wild-type.

## Introduction

Lung cancer is one of the most devastating illnesses, responsible for most cancer-related deaths. It is divided into small cell lung cancer (SCLC) and non-small cell lung cancer (NSCLC) based on histology; the latter accounts for 85% of all lung cancer cases (1). Lung cancer patients often develop brain metastases (BMs). BMs are roughly ten times more prevalent than their primary counterpart (2), and lung cancer accounts for the majority of cases of BMs (3). The incidence of BMs at the time of initial lung cancer diagnosis is over 25% and 20% within a year of primary tumor diagnosis (4, 5). BMs are associated with poor prognosis and are considered challenging to treat because of their unique blood-brain barrier (BBB) (6), making local therapy such as radiation therapy and surgery upfront choices for treating BMs. Per the current guidelines, surgery should be offered to the population with large tumors with mass effect, and stereotactic radiosurgery (SRS) alone is recommended in patients with limited brain metastases (7). However, SRS alone is the standard therapy for patients with 1-4 BMs but also can be considered for patients with more than 4 lesions (8–10). SRS, whole brain radiation therapy, or their combination are reasonable options for other patients. Nonetheless, SRS is preferred because it is well known to preserve neurocognitive function, as neurocognitive impairment is the biggest concern of patients and clinicians.

Further, immunotherapy (IT) has revolutionized the treatment approach in patients with advanced cancers. A study reported that 44% of the metastatic cancer population was eligible for IT (11). IT has become the first-line therapy in metastatic NSCLC, predominantly in wild-type or non-targetable mutation cases (12). IT has been reported to have a considerable response in advanced-stage NSCLC patients (13–15). The growing use of IT in metastatic cancer has led to more and more patients receiving the combined IT and SRS for BMs. However, the right sequence and right timing of IT relative to SRS are still unclear. Therefore, it has become more crucial to explore the determinants of IT responsiveness and its interaction with the SRS to determine the right timing and sequence for IT.

With growing insight into the IT response determinants, traditionally used prognostic indices may not be equally efficient in predicting OS for every BM patient. At present, there are several established prognostic grading indexes for BM, including the recursive partitioning analysis (RPA), disease-specific graded prognostic assessment (DS-GPA), score index for radiosurgery (SIR), and a basic score for brain metastases (BS-BM) which comprised of some or all of the following parameters: Karnofsky Performance Status (KPS), age, extra-cranial disease status, primary diagnosis, number of BMs, mutation status, and PD-L1 expression status (16–18). Cumulative tumor volume is an important prognostic factor; its prognostic importance has previously been reported in different cancers (19–21). Despite that, it is not included in any traditionally used prognostic indexes. On the other hand, PD-L1 expression in cancer is found to be transient and shows topographic heterogeneity, creating uncertainty about it as a biomarker for predicting IT effectiveness (22). Moreover, the dynamics of the PD-1 and PD-L1 axis might change subject to the type of therapy and sequence of administration (23, 24). However, it has been given immense importance when predicting prognosis. Hence, there is an imperative need for an up-to-date prognostic assessment model.

In line with evolving insights into determinants of IT responsiveness and SRS role in reshaping tumor immune microenvironment, we hypothesize that the use of combined IT and SRS in treating NSCLCBM can be more beneficial than SRS alone, and there is an optimal window for initiating IT in relation to SRS. Peri-SRS IT and CITV might serve as prognosis predictors.

## Materials and methods

### Study population and data acquisition

This study was approved by the Guangdong Sanjiu brain hospital ethical committee. Due to the retrospective nature of the cohort, the requirement to obtain informed consent was waived. In total, 86 NSCLCBM patients who received SRS between January 2018 and September 2021 were included. First, the data of 43 patients with NSCLCBM who received SRS and IT for BM were extracted from our hospital’s electronic medical records. Then, a propensity-score matching (PSM) analysis (selecting covariates = age, gender, KPS, and mutation status; method = nearest; caliper = 0.05) was performed to find the closest matched 43 patients from a pool of over 200 NSCLCBM patients in whom IT was not used but SRS. To be included in the present cohort, patients had to be histologically proven NSCLC, BM diagnosed with contrast-enhanced MRI, and had undergone SRS for BM. The endpoint of the current cohort was the death or last follow-up, whichever occurred first; to overcome time bias, two zero times were selected, one from the day of the first SRS and the second from the day of the initial BM diagnosis. The OS was calculated from the initial BM diagnosis and first SRS to the death or last follow-up. Follow-up data were collected retrospectively from the electronic medical database, treating physicians or patients to diminish missing data.

### Patients’ characteristics

Detailed data of each patient, including demographic, clinical, and laboratory parameters, were collected from the electronic medical record upon admission for undergoing SRS for BMs. If some laboratory tests were done more than once, a test reading closest to the date of the initial SRS was recorded. Continuous predictors such as age, CITV, number of tumors, lactate dehydrogenase (LDH), neutrophil-lymphocyte ratio (NLR), neuron-specific enolase (NSE), carcinoembryonic antigen (CEA), and squamous cell carcinoma (SCC) antigen were classified into 2 groups. The cutoffs for age, LDH, NLR, NSE, CEA, SCC, CITV, and the number of BMs were 60 years, 250 U/L, 3.0, 16.3 ng/ml, 5 ng/ml, 1.5 ng/ml, 4 cm^3^, and 3 BMs, respectively. They were analyzed as categorical variables. The CITV was defined as the sum of the tumor volume of all treated BM lesions. Contrast-enhanced MRI was used to evaluate BMs.

### Stereotactic radiosurgery parameters

Patients were treated either by single-SRS or fractionated-SRS by the Novalis Tx® system (BrainLAB AG, Feldkirchen, Germany; Varian Medical System, Palo Alto, CA, USA). Dosage and fraction details are presented in **Table 1**. In the case of fractionated-SRS, fractions were delivered with a gap of 1–3 days. Supportive measures included regular administration of mannitol after SRS, contraindicated otherwise.

**Table 1:** Patients' baseline clinicodemographic characteristics.

Characteristics	SRS with IT (N = 43) n (%)	SRS without IT (N = 43) n (%)	P-value
Gender			
Female	3 (7)	3 (7)	1
Male	40 (93)	40 (93)	
Age (years)			
≤60	24 (45.8)	21 (48.8)	0.666
>60	19 (44.2)	22 (51.2)	
Median (IQR)	59 (49-65)	61 (56-66.5)	
Smoking			
Smoker	24 (45.8)	20 (46.5)	0.518
Non-smoker	19 (44.2)	23 (53.5)	
KPS			
90	9 (20.9)	6 (14)	0.813
80	12 (27.9)	13 (30.2)	
70	11 (25.6)	13 (30.2)	
60	7 (16.3)	9 (20.9)	
50	3 (7)	2 (4.7)	
40	1 (2.3)	0 (0)	
Median (IQR)	70 (65-80)	70 (65-80)	
Comorbidities			
Present	11 (25.6)	17 (39.5)	0.250
Absent	32 (74.4)	26 (60.5)	
Histology			
Adenocarcinoma	31 (72.1)	39 (90.7)	0.075
Squamous cell carcinoma	11 (25.6)	4 (9.3)	
Sarcomatoid carcinoma	1 (2.3)	0 (0)	
Mutation status			
EGFR/ROS1/ALK mutation	6 (14)	6 (14)	0.854
Wild type	37 (86)	27 (62.8)	
Unknown	0 (0)	10 (23.2)	
Extra-cranial metastases			
Present	24 (45.8)	21 (48.8)	0.666
Absent	19 (44.2)	22 (51.2)	
Extra-cranial metastasis control			
Controlled	12 (27.9)	11 (25.6)	0.286
Uncontrolled	11 (25.6)	6 (14)	
Non-applicable	16 (37.2)	24 (55.8)	
Unknown	4 (9.3)	2 (4.6)	
Leptomeningeal metastases			
Present	7 (16.3)	2 (4.7)	0.159
Absent	36 (83.7)	41 (95.3)	
TNM classification 8^th^			
**T stage**			
T1	1 (2.3)	5 (11.6)	0.181
T2	9 (20.9)	14 (32.6)	
T3	8 (18.6)	7 (16.3)	
T4	13 (30.2)	8 (18.6)	
Unknown	12 (28)	9 (20.9)	
**N stage**			
N1	5 (11.6)	1 (2.3)	0.076
N2	13 (30.2)	20 (46.5)	
N3	19 (44.2)	13 (30.2)	
Unknown	6 (14)	9 (21)	
**M stage**			
M1b	10 (23.3)	8 (18.6)	0.791
M1c	33 (76.7)	35 (81.4)	
RPA score			
1	3 (7)	6 (14)	0.538
2	30 (69.7)	29 (67.4)	
3	10 (23.3)	8 (18.6))	
Lung-molGPA index			
0.5	1 (2.3)	4 (9.3)	0.514
1	7 (16.3)	8 (18.6)	
1.5	8 (18.6)	4 (9.3)	
2	10 (23.3)	12 (27.9)	
2.5	7 (16.3)	9 (20.9)	
3	9 (20.9)	6 (14)	
3.5	1 (2.3)	0 (0)	
Number of BMs			
≤3	32 (74.4)	30 (69.7)	0.81
>3	11 (25.6)	13 (30.3)	
Total number of BMs	135	133	
Median (IQR)	2 (1-3.5)	2 (1-4.5)	
Cumulative intracranial tumor volume (cm^3^)			
≤4	15 (34.9)	10 (23.3)	0.273
>4	26 (60.5)	33 (76.7)	
Unknown	2 (4.6)	0 (0)	
Median (IQR)	5.7 (2-14.8)	11.2 (4.55-22.7)	
Distribution of BMs			
Supra-tentorium	24 (55.8)	22 (51.1)	0.665
Infra-tentorium	5 (11.6)	8 (18.6)	
Both	14 (32.6)	13 (30.3)	
LDH (U/L)			
≤250	18 (41.8)	26 (60.4)	0.118
>250	12 (27.9)	6 (14)	
Unknown	13 (30.3)	11 (25.6)	
Median (IQR)	239.6 (206.3-271.7)	198.1 (161-221)	
NLR			
≤3.0	10 (23.3)	11 (25.6)	1
>3.0	33 (76.7)	32 (74.4)	
Median (IQR)	4.1 (3.1-6.45)	4.2 (3.1-5.85)	
NSE (ng/ml)			
≤16.3	21 (48.8)	25 (58.1)	0.177
>16.3	21 (48.8)	12 (27.9)	
Unknown	1 (2.4)	6 (14)	
Median (IQR)	15.2 (11.1-21.3)	14.9 (11.8-19.7)	
CEA ( ng/ml)			
≤5	24 (55.8)	24 (55.8)	0.975
>5	19 (44.2)	17 (39.5)	
Unknown	0 (0)	2 (4.7)	
Median (IQR)	4.1 (2.4-21)	4.3 (2.7-13.1)	
SCC antigen ( ng/ml)			
≤1.5	29 (67.4)	30 (69.7)	1
>1.5	7 (16.3)	6 (14)	
Unknown	7 (16.3)	7 (16.3)	
Median (IQR)	0.8 (0.4-1.3)	0.9 (0.6-1.3)	
Radiation type			
Single-fraction SRS dose (Gy)			
15	1 (2.4)	0 (0)	
16	15 (34.8)	12 (27.9)	
18	13 (30.2)	9 (20.9)	
Multi-fraction SRS dose(Gy)/fraction, total fractions			
8/F, 3F	9 (20.9)	9 (20.9)	
10/F, 2F	4 (9.3)	12 (27.9)	
11/F, 2F	1 (2.4)	1 (2.4)	

SRS, stereotactic radiosurgery; IT, immunotherapy; IQR, interquartile range; KPS, Karnofsky performance status; T, tumor; N, node; M, metastasis; RPA, recursive partitioning analysis; Lung-molGPA, molecular graded prognostic assessment for lung cancer; BMs, brain metastases; LDH, lactate dehydrogenase; NLR, neutrophil-lymphocyte ratio; NSE, neuron-specific enolase; CEA, carcinoembryonic antigen; SCC, squamous cell carcinoma; FSRS, fractionated stereotactic radiosurgery; Gy, gray; F, fraction.

### Construction of prognosis prediction model

Cox proportional hazards regression analysis was used to construct a prognosis assessment model. A P-value of a variable had to be ≤ 0.15 in univariable analysis to fit in the multivariable analysis. The nomogram was established utilizing the rms package in R. The concordance index (C-index), calibration curves, and receiver operating characteristic (ROC) curves were implemented to determine the predictive model accuracy.

### Statistical analysis

Descriptive statistics for quantitative variables were demonstrated as medians (interquartile range, IQR) and categorical variables as numbers (percentages, %). Wilcoxon rank sum test or Kruskal Wallis rank sum test was conducted for continuous variables, and categorical variables were analyzed *via* Chi-square or Fisher’s exact tests, as appropriate. OS was estimated using Kaplan–Meier analysis, and a log-rank test evaluated the differences between groups. Cox regression was utilized to identify significant prognostic factors. Hazard ratios (HRs) are presented with 95% confidence intervals (CIs). All analyses were done using R version 4.1.2 (http://www.R-project.org). P-value was considered statistically significant if it was <0.05.

## Results

### Patient cohort

The data of 86 NSCLCBM patients treated with SRS to 268 BMs were retrospectively obtained from the Guangdong Sanjiu Brain Hospital database of the patients treated for BMs between January 2018 and September 2021. The median follow-up time was 11.3 months (IQR, 6.8 - 21.4) and 8.5 (IQR, 4.2 - 15.8) following BM diagnosis and SRS, respectively. Of 86, 51 (59.3%) were diagnosed with BM at the time of primary tumor diagnosis (≤ 2 weeks); 11 patients were alive at the time of analysis. The patients’ median age was 60 years (age range 28–80 years), of whom 80 (80/86, 93%) were males. Most patients (70/86, 81.4%) had adenocarcinoma histology, followed by squamous cell carcinoma (15/86, 17.4%) and sarcomatoid carcinoma (1/86, 1.2%). Most patients (64/86, 74.4%) were wild-type, 12 (14%) harbored an EGFR/ALK/ROS1 mutation, and 10 (11.6%) had unknown mutation status. Based on treatment, patients were stratified into two groups, “SRS with IT” (n=43, 50%) and “SRS without IT” (n=43, 50%). No significant discrepancies were found in patients’ clinicodemographic characteristics between the two groups (**Table 1**).

### Overall survival

**Figure 1:**
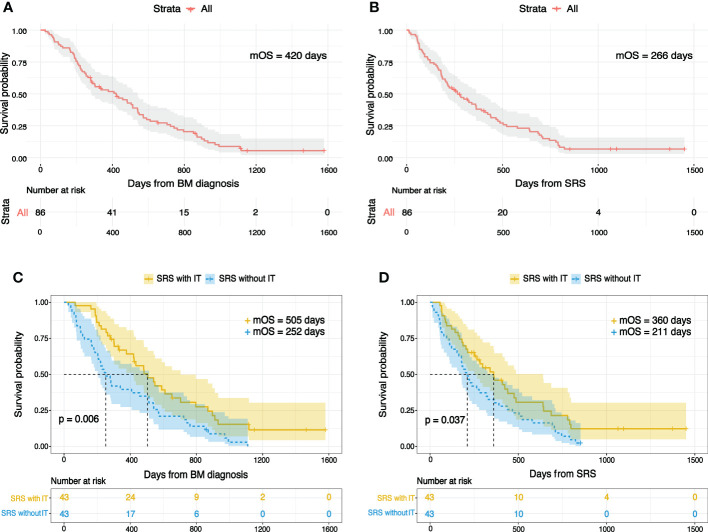
Kaplan-Meier overall survival plots for the entire study population following **(A)** BM diagnosis **(B)** SRS. Overall survival for individuals treated by SRS with IT or SRS without IT from **(C)** BM diagnosis **(D)** SRS.

The median OS for the entire population was 420 days (14 months) and 266 days (8.7 months) from the date of BM diagnosis and SRS, respectively (**Figures 1A, B**). The log-rank test identified a significant difference in OS between SRS with IT and SRS without IT groups (**Figures 1C, D**). Patients treated with IT had better OS than those in whom IT wasn’t used. The median OS for the IT group from the day of BM diagnosis was 505 days (16.8 months) and 252 days (8.4 months) for no IT group. OS for the IT group from the day of SRS was 360 days (12 months) and 211 days (7 months) for no IT group. Univariate analysis revealed several clinical factors were potentially associated with OS (**Table 2**). IT and RPA were the independent prognostic factors in multivariate analysis, whereas CITV was marginally significant. However, SCC antigen tumor marker was significant, and NLR was marginally significant only when the OS was calculated from BM diagnosis.

**Table 2:** Univariate and multivariate analysis for overall survival.

Variables	From BM diagnosis		From SRS	
	HR (95% CI)	P-value	HR (95% CI)	P-value
Univariate analysis				
Sex	1.1 (0.474 - 2.552)	0.824	0.827 (0.357 - 1.918)	0.658
Age	1.097 (0.697 - 1.727)	0.689	1.004 (0.635 - 1.59)	0.985
Smoking	1.273 (0.803 - 2.019)	0.305	1.347 (0.851 - 2.133)	0.203
Histology	0.974 (0.733 - 1.293)	0.854	0.948 (0.716 - 1.257)	0.712
Mutation	0.656 (0.343 - 1.254)	0.202	0.688 (0.362 - 1.308)	0.254
Extra-cranial Metastasis	1.823 (1.141 - 2.912)	0.012	1.618 (1.02 - 2.565)	0.041
Leptomeningeal Metastasis	0.837 (0.400 - 1.751)	0.637	0.918 (0.439 - 1.922)	0.821
T stage	1.029 (0.771 - 1.374)	0.845	1.084 (0.820 - 1.433)	0.571
N stage	1.116 (0.749 - 1.663)	0.59	1.109 (0.754 - 1.633)	0.599
M stage	0.954 (0.523 - 1.741)	0.879	0.961 (0.526 - 1.753)	0.896
RPA	1.88 (1.216 - 2.907)	0.004	1.945 (1.282 - 2.952)	0.001
molGPA	0.683 (0.496 - 0.943)	0.020	0.724 (0.530 - 0.99)	0.043
KPS	0.981 (0.961 - 1.001)	0.057	0.983 (0.963 - 1.003)	0.09
CITV (cm^3^)	0.56 (0.335 - 0.936)	0.027	0.679 (0.409 - 1.129)	0.136
Distribution of BMs	1.1 (0.851 - 1.422)	0.466	1.052 (0.815 - 1.358)	0.698
Number of BMs	0.618 (0.377 - 1.013)	0.056	0.678 (0.414 - 1.109)	0.122
Immunotherapy	0.527 (0.332 - 0.838)	0.006	0.617 (0.39 - 0.975)	0.038
NLR	1.528 (0.896 - 2.606)	0.12	1.379 (0.810 - 2.347)	0.236
LDH	1.352 (0.723 - 2.527)	0.345	1.299 (0.708 - 2.384)	0.398
NSE	1.635 (0.997 - 2.682)	0.051	1.803 (0.098 - 2.961)	0.020
CEA	0.864 (0.541 - 1.379)	0.54	0.866 (0.543 - 1.382)	0.547
SCC antigen	2.193 (1.103 - 4.358)	0.025	2.746 (1.353 - 5.574)	0.005
Multivariate analysis				
Immunotherapy	0.363 (0.199 - 0.661)	<0.001	0.472 (0.260 - 0.857)	0.014
Extra-cranial Metastasis	1.374 (0.617 - 3.06)	0.436	0.918 (0.419 - 2.008)	0.830
RPA	2.219 (1.163 - 4.235)	0.016	2.562 (1.340- 4.900)	0.004
molGPA	1.047 (0.571 - 1.919)	0.882	1.051 (0.553- 1.998)	0.878
KPS	1.012 (0.976 - 1.049)	0.517	1.028 (0.991 - 1.066)	0.138
CITV (cm^3^)	1.716 (0.922 - 3.196)	0.089	1.783 (0.941 - 3.376)	0.076
Number of BMs	0.673 (0.326 - 1.390)	0.285	0.579 (0.269 - 1.245)	0.162
NSE	1.171 (0.557 - 2.458)	0.677	1.504 (0.742 - 3.045)	0.257
SCC antigen	2.542 (1.112 – 5.810)	0.027	1.917 (0.847 - 4.337)	0.118
NLR	1.856 (0.976 - 3.530)	0.060		

T, tumor; N, node; M, metastasis; RPA, recursive partitioning analysis; Lung-molGPA, molecular graded prognostic assessment for lung cancer; KPS, Karnofsky performance status; CITV, cumulative intracranial tumor volume; BMs, brain metastases; NLR, neutrophil-lymphocyte ratio; LDH, lactate dehydrogenase; NSE, neuron-specific enolase; CEA, carcinoembryonic antigen; SCC, squamous cell carcinoma; SRS, stereotactic radiosurgery.

### Optimal sequencing and timing of IT in relation to SRS

In total, 43 patients who received IT for BMs at any point during the disease course were divided into three groups by IT timing to SRS. If the IT was initiated more than a month before the date of SRS, they were allocated to the IT before SRS group; if IT was administrated within a month before or after the day of SRS or started more than a month before but continued at the time of SRS were assigned to the concurrent group; if IT was started more than a month after the day of SRS were allotted to the IT after SRS group. If patients have undergone FSRS, the start date was considered the zero time. The number of patients who received IT before, concurrent, and after were 9, 17, and 17, respectively. The patients’ median age was 59 years (range 29-79 years). Of 43, 40 (93%) were males. Patients’ relevant characteristics are presented in **Table 3**. None of the characteristics were significantly different between the three groups.

**Table 3:** Relevant characteristics of NSCLCBM patients treated with SRS plus IT.

Characteristics	IT before SRS (n = 9) n (%)	Concurrent (n = 17) n (%)	IT after SRS (n = 17) n (%)	P value
Histology				
Adenocarcinoma	5 (55.6)	11 (64.7)	15 (88.3)	0.202
Squamous cell carcinoma	4 (44.4)	5 (29.4)	2 (11.7)	
Sarcomatoid carcinoma	0 (0)	1 (5.9)	0 (0)	
Mutation status				
Wild type	8 (88.9)	14 (82.4)	15 (88.3)	1
EGFR/ROS1/ALK mutation	1 (11.1)	3 (17.6)	2 (11.7)	
PD-L1 expression				
>50%	1 (11.1)	2 (11.8)	0 (0)	0.423
1-50%	1 (11.1)	3 (17.6)	3 (17.6)	
<1%	0 (0)	4 (23.5)	4 (23.5)	
unknown	7 (77.8)	8 (47.1)	10 (58.8)	
KPS				
90	1 (11.1)	4 (23.5)	4 (23.5)	0.527
80	1 (11.1)	5 (29.5)	6 (35.3)	
70	3 (33.4)	4 (23.5)	4 (23.5)	
60	2 (22.2)	4 (23.5)	1 (5.9)	
50	1 (11.1)	0 (0)	2 (11.8)	
40	1 (11.1)	0 (0)	0 (0)	
Median (IQR)	70 (60-70)	80 (70-80)	80 (70-80)	
Extra-cranial metastases				
Present	7 (77.8)	7 (41.2)	10 (58.8)	0.202
Absent	2 (22.2)	10 (58.8)	7 (41.2)	
Number of BMs				
≤3	5 (55.6)	15 (88.2)	12 (70.6)	0.191
>3	4 (44.4)	2 (11.8)	5 (29.4)	
Total number of BMs (median)	37	39	57	
Median (IQR)	3 (1-4)	1 (1-3)	2 (1-4)	
CITV (cm^3^)				
≤4	3 (33.3)	6 (35.3)	6 (35.3)	1
>4	5 (55.6)	11 (64.7)	10 (58.8)	
Unknown	1 (11.1)	0 (0)	1 (5.9)	
Median (IQR)	7.3 (2.6-13.3)	7.5 (1.8-14.8)	5.7 (3.1-11.4)	
Immunotherapeutic agents				
Nivolumab (PD-1); Pembrolizumab (PD-1); Sintilimab (PD-1); Toripalimab (PD-1); Tislelizumab (PD-1); Camrelizumab (PD-1); KN046 (PD-L1 and CTLA-4)				
IT cycles				
Median (range)	6.5 (2-14)	4 (1-19)	7 (1-28)	
Timing of IT (months)				
Median (range)	4.4 (1.2-15.5)	–	6.3 (1.8-16.4)	
Radiation type				
Single-fraction SRS dose (Gy)				
15	1 (11.1)	0 (0)	0 (0)	
16	3 (33.4)	7 (41.2)	5 (29.4)	
18	2 (22.2)	4 (23.5)	7 (41.1)	
Multi-fraction SRS dose(Gy)/fraction, total fractions				
8/F, 3F	2 (22.2)	5 (29.4)	2 (11.8)	
10/F, 2F	1 (11.1)	1 (5.9)	2 (11.8)	
11/F, 2F	0 (0)	0 (0)	1 (5.9)	
irAE (CTCAE)				
Skin	1 (11.1)	1 (5.9)	–	
Lung	1 (11.1)	–	–	
Gastro-intestinal tract	–	1 (5.9)	–	
RTOG Radiation Toxicity Grading (grade 3 onward)				
RTOG acute radiation morbidity	–	–	–	
RTOG late radiation morbidity	–	–	–	

IT, immunotherapy; SRS, stereotactic radiosurgery; BM, brain metastases; IQR, interquartile range; KPS, Karnofsky performance status; CITV, cumulative intracranial tumor volume; FSRS, fractionated stereotactic radiosurgery; Gy, gray; F, fraction; irAE, immune-related adverse events; RTOG, Radiation Therapy Oncology Group; CTCAE, common terminology criteria for adverse events.

**Figure 2:**
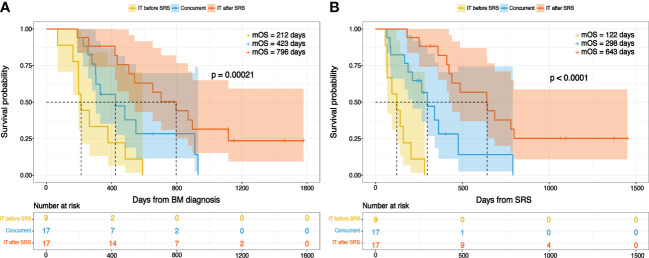
Overall survival with reference to immunotherapy timing from the day of **(A)** BM diagnosis **(B)** SRS.

As per our analysis, the recipient of IT after SRS had an excellent OS, and those who received IT before SRS had the worst OS (**Figures 2A, B**). The median OS for those in whom IT was initiated after SRS was 796 days (26.5 months) from the day of BM diagnosis and 643 days (21.4 months) from SRS, which was the highest among the three groups. Recipient of IT before SRS had a median OS of 212 days (7.1 months) following BM diagnosis and 122 days (4.1 months) from SRS, which was the worst among the three groups. Individuals who received IT and SRS concurrently showed a median OS of 423 days (14.1 months) and 298 days (9.9 months) from the date of BM diagnosis and SRS, respectively. In univariate analysis, we found several clinical factors were potentially associated with OS. Multivariate analysis identified that the peri-SRS timing of IT administration was the significant independent prognostic factor for OS, as shown in **Table 4**. Smoking was a significant survival predictor when OS was calculated from the date of BM diagnosis.

**Table 4:** Univariate and multivariate analysis for overall survival in NSCLCBM patients treated with SRS plus IT.

Variables	From BM diagnosis		From SRS	
	HR (95% CI)	P-value	HR (95% CI)	P-value
Univariate analysis				
Sex	1.91 (0.574 - 6.353)	0.292	1.242 (0.374 - 4.129)	0.723
Age	1.269 (0.635 - 2.537)	0.501	1.051 (0.517 - 2.133)	0.891
Smoking	1.697 (0.835 - 3.449)	0.144	1.6 (0.791 - 3.236)	0.191
Histology	0.986 (0.717 - 1.356)	0.931	0.971 (0.71 - 1.33)	0.856
Mutation	0.718 (0.273 - 1.889)	0.502	0.701 (0.269 - 1.826)	0.467
Extra-cranial Metastasis	2.027 (0.963 – 4.264)	0.063	1.719 (0.843 - 3.506)	0.136
Leptomeningeal Metastasis	0.766 (0.313 - 1.871)	0.558	0.931 (0.382 - 2.271)	0.876
T stage	1.745 (1.004 - 3.033)	0.048	1.858 (1.078 – 3.201)	0.026
N stage	1.206 (0.692 - 2.102)	0.509	1.246 (0.728 - 2.134)	0.422
M stage	1.457 (0.560 - 3.791)	0.44	1.343 (0.516 - 3.493)	0.546
RPA	1.786 (0.848 - 3.762)	0.127	1.482 (0.744 - 2.954)	0.264
molGPA	0.747 (0.468 – 1.192)	0.221	0.817 (0.521 - 1.281)	0.378
KPS	0.987 (0. 958 - 1.014)	0.313	0.993 (0.966 - 1.02)	0.6
CITV (cm^3^)	0.683 (0.326 - 1.429)	0.311	1.294 (0.614 - 2.726)	0.498
Distribution of BMs	1.016 (0.691 - 1.495)	0.935	0.860 (0.579 - 1.277)	0.454
Number of BMs	0.589 (0.278 - 1.248)	0.167	0.782 (0.369 - 1.656)	0.521
Immunotherapy timing	0.397 (0.243 - 0.648)	0.0002	0.206 (0.112 - 0.381)	<0.001
NLR	1.664 (0.719 - 3.848)	0.234	1.59 (0.689 - 3.674)	0.277
LDH	1.974 (0.794 - 4.905)	0.143	1.832 (0.773 - 4.345)	0.169
NSE	1.644 (0.815 - 3.314)	0.165	1.854 (0.915 - 3.756)	0.087
CEA	0.965 (0.484 - 1.923)	0.919	0.894 (0.447 - 1.788)	0.752
SCC antigen	1.922 (0.699 - 5.287)	0.206	2.542 (0.867 - 7.451)	0.089
Multivariate analysis				
Immunotherapy timing	0.132 (0.034 - 0.517)	0.004	0.14 (0.044 - 0.450)	0.001
Extra-cranial Metastasis	4.704 (0.737 - 30.026)	0.102	0.587 (0.166 - 2.074)	0.408
Smoking	8.169 (1.417 - 47.082)	0.019		
RPA	0.492 (0.138 - 1.754)	0.274		
T stage	0.887 (0.385 - 2.047)	0.779	1.77 (0.862 - 3.634)	0.120
NSE			1.989 (0.617 - 6.416)	0.25
SCC antigen			0.442 (0.080 - 2.428)	0.347
LDH	1.787 (0.493 - 6.473)	0.377		

BMs, brain metastases; SRS, stereotactic radiosurgery; T, tumor; N, node; M, metastasis; RPA, recursive prognostic assessment; Lung-molGPA, molecular graded prognostic assessment for lung cancer; KPS, Karnofsky performance status; CITV, cumulative intracranial tumor volume; NLR, neutrophil-lymphocyte ratio; LDH, lactate dehydrogenase; NSE, neuron-specific enolase; CEA, carcinoembryonic antigen; SCC, squamous cell carcinoma.

### Toxicity

The adverse events related to combined IT and SRS were manageable and consistent with previous publications (25, 26). In our analysis, 4 patients (4/43, 9.3%) appeared to have grade 3 immune-related adverse events, 2 (2/43, 4.65%) had skin complications, 1 (1/43, 2.3%) lung, and 1 (1/43, 2.3%) gastrointestinal tract complication. All patients recovered utterly within a few days of treatment. No patient exhibited grade 4 or 5 toxicity. Moreover, no grade 3 onward radiation toxicity was reported based on the data obtained from our hospital’s electronic medical database, treating physicians, and patients (**Table 3**).

### Prognostic nomogram

**Figure 3:**
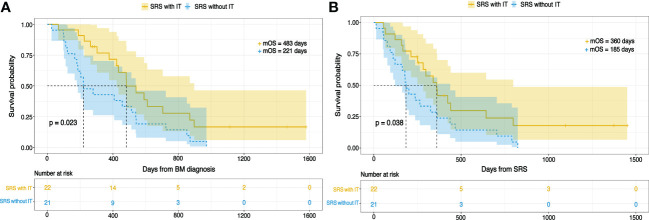
The Kaplan-Meier curves for OS in NSCLCBM patients treated by SRS with IT or SRS without IT from **(A)** BM diagnosis **(B)** SRS.

The entire study population was stratified into the training set and validation set by random number generator using R software (total population = 86, training cohort = 43, validation cohort = 43). In the training cohort, the number of patients received IT was 22, and 21 didn’t receive IT. Kaplan-Meier analysis revealed OS was significantly different between SRS with IT and SRS without IT groups in the training cohorts. The patient who received IT had improved OS than those who didn’t, as shown in **Figures 3A, B**. The median OS for the IT group from the day of BM diagnosis was 483 days (16.1 months) and 221 days (7.4 months) for the no IT group. OS for the IT group from the day of SRS was 360 days (12 months) and 185 days (6.2 months) for no IT group.

**Figure 4:**
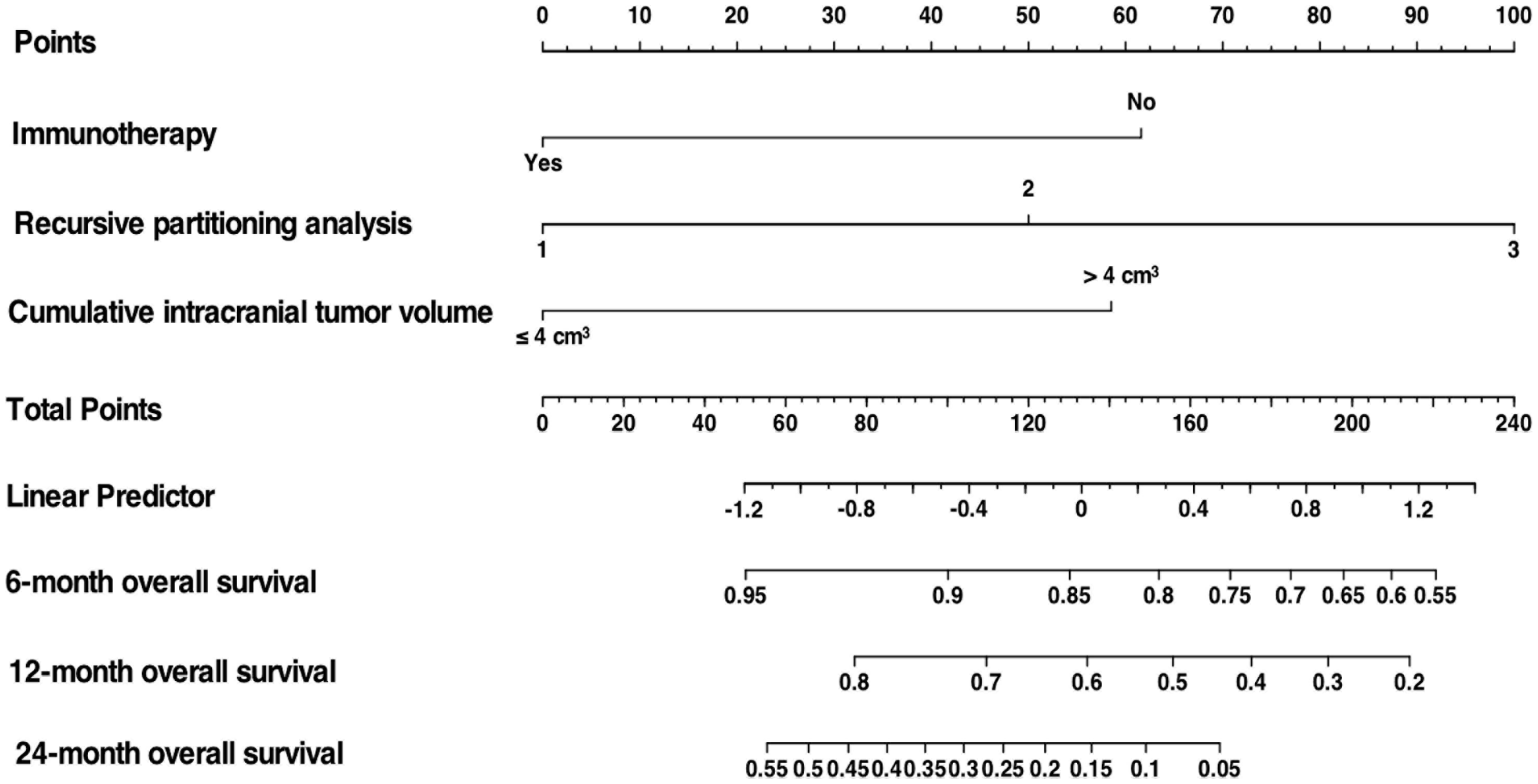
Nomogram to predict OS in NSCLCBM patients treated with SRS based on 3 independent prognostic factors identified in the training cohort.

The clinicodemographic and laboratory variables of the patients in the training set were evaluated to develop the prognosis prediction model. A univariate cox regression analysis was conducted to explore possible variables related to the survival in the training cohort population. Unifactorial analysis indicated several potential prognosis predictors (**Table 5**). Multivariate cox regression analysis with stepwise forward selection was run, integrating significant variables in the univariate analyses, revealing that IT, RPA, and CITV were the significant independent prognostic factors. By integrating these significant independent prognostic variables, we developed a nomogram for predicting OS in NSCLCBM patients treated with SRS (**Figure 4**).

**Table 5:** Univariate and multivariate analysis for overall survival.

Variables	Training cohort (n = 43)		Validation cohort (n = 43)	
	HR (95% CI)	P-value	HR (95% CI)	P-value
Univariate analysis				
Sex	0.820 (0.248 - 2.705)	0.744	0.677 (0.205 - 2.236)	0.522
Age	0.919 (0.479 - 1.764)	0.8	1.920 (0.480 - 1.765)	0.801
Smoking	0.957 (0.499 - 1.834)	0.894	1.014 (0.531 - 1.937)	0.966
Histology	1.911 (0.771 - 4.733)	0.162	1.981 (0.787 - 4.986)	0.147
Mutation	0.724 (0.652 - 2.469)	0.505	0.850 (0.329 - 2.197)	0.737
Extra-cranial Metastasis	1.269 (1.141 - 2.912)	0.483	1.364 (0.698 - 2.667)	0.364
Leptomeningeal Metastasis	0.876 (0.307 - 2.498)	0.804	1.012 (0.356 - 2.878)	0.982
T stage	0.858 (0.575 - 1.28)	0.452	0.942 (0.644 - 1.379)	0.76
N stage	1.027 (0.642 - 1.643)	0.991	1.026 (0.657 - 1.603)	0.91
M stage	0.45 (0.170 - 1.188)	0.107	0.716 (0.285 - 1.8)	0.477
RPA	1.636 (0.843 - 3.174)	0.145	1.859 (0.978 - 3.537)	0.059
molGPA	0.832 (0.521 - 1.329)	0.442	0.810 (0.521 - 1.262)	0.352
KPS	0.998 (0.970 - 1.026)	0.864	0.994 (0.967 - 1.022)	0.671
CITV (cm^3^)	1.947 (0.880 - 4.306)	0.1	1.942 (0.877 - 4.301)	0.102
Distribution of BMs	0.896 (0.617 - 1.302)	0.565	0.882 (0.611 - 1.272)	0.501
Number of BMs	0.690 (0.340 - 1.4)	0. 303	0.753 (0.371 - 1.528)	0.432
Immunotherapy	0.475 (0.246 - 0.915)	0.026	0.505 (0.262 - 0.974)	0.042
NLR	1.637 (0.832 - 3.222)	0.154	1.238 (0.631 - 2.428)	0.534
LDH	0.999 (0.997 - 1.002)	0.673	0.999 (0.996 - 1.002)	0.461
NSE	1.135 (0.559 - 2.303)	0.726	1.135 (0.560 - 2.299)	0.725
CEA	0.999 (0.996 - 1.001)	0.217	0.999 (0.996 - 1.001)	0.216
SCC antigen	1.529 (0.519 - 4.501)	0.441	1.432 (0.480 - 4.273)	0.52
Multivariate analysis				
Immunotherapy	0.412 (0.208 - 0.818)	0.011	0.267 (0.117 - 0.610)	0.002
RPA	2.064 (1.064 - 4.001)	0.032	3.069 (1.471- 6.402)	0.003
Univariate analysis				
CITV (cm^3^)	2.486 (1.108 - 5.576)	0.027	2.334 (1.018 - 5.353)	0.045
M stage	0.409 (0.151 - 1.109)	0.079		
Histology			2.462 (0.862 - 7.034)	0.092

T, tumor; N, node; M, metastasis; RPA, recursive prognostic assessment; Lung-molGPA, molecular graded prognostic assessment for lung cancer; KPS, Karnofsky performance status; CITV, cumulative intracranial tumor volume; BMs, brain metastases; NLR, neutrophil-lymphocyte ratio; LDH, lactate dehydrogenase; NSE, neuron-specific enolase; CEA, carcinoembryonic antigen; SCC, squamous cell carcinoma.

## Discussion

Theoretically, PD-L1 expression level, neoantigen, microsatellite, tumor mutation burden, tumor heterogeneity, tumor-infiltrating lymphocytes, regulatory T cells, and damage-associated molecular patterns (DAMPs) are well-known determinants of IT responsiveness (27). These determinants can be modulated by radiotherapy, immunogenic chemotherapeutic agents, and combination therapies. Radiotherapy is widely known as an inducer of immunogenic cell death. In addition, radiotherapy can increase PD-L1 expression level (23), inhibit T regulatory cells, release antigens and damage-associated molecular patterns from cancer cells (27), increase BBB permeability (28–30), and convert immunologically cold tumors to hot ones (31). Furthermore, radiotherapy can also affect non-treatment lesions when combined with IT, known as an abscopal effect (32). Immunogenic chemotherapeutic agents not only sensitize previously resistant but also enhance the response of IT by increasing tumor-infiltrating lymphocytes (33). Taken together, the right combination, right sequence, and right timing can improve IT response even in tumors known to be intrinsically resistant.

In line with theoretical knowledge, our analysis revealed that combined use of IT and SRS was associated with OS in NSCLCBM patients (from BM diagnosis: HR 0.363, 95% CI 0.199 - 0.661, *P* < 0.001; from SRS: HR 0.472, 95% CI 0.260 - 0.857, *P* = 0.014). Individuals receipt IT in combination with SRS had better OS than those who didn’t receive IT (from the day of BM diagnosis: 16.8 vs. 8.4 months, P = 0.006; from the day of SRS: 12 vs. 7 months, P = 0.037), which is consistent with the studies conducted by Abdulhaleem et al. (40 vs. 8 months), Lanier and colleagues (15.9 vs. 6.1 months), and Chen and partners (19.4 vs. 12.9 months) (34–36). Similarly, Enright and colleagues insisted that SRT plus immune checkpoint inhibitors (ICIs) improved long-term survival in NSCLCBM patients compared to SRT alone (37). Meanwhile, there was no increase in adverse events, which is consistent with present cohort findings. Moreover, Stokes et al. (10.8 vs. 6.1 months), Diao et al. (15.1 vs. 7.8 months), Knisely et al. (21.3 vs. 4.9 months), Gabani et al. (11.1 vs. 6.2 months), and Kaidar-Person et al. (15 vs. 6 months) also reported similar results in melanoma patients (38–42). Taken together, we can conclude that IT can be a powerful tool when combined with radiotherapy.

Despite the growing evidence of the positive synergy of radiotherapy and IT, the optimal window for initiating IT relative to SRS remains unclear. Intuitively, delay of specific time duration in IT administration post-SRS can be beneficial for allowing some time to get the immune system in action (release antigen from dying cancer cells, their recognition and processing by antigen-presenting cells, and delivery to T cells) and early and delayed effects of radiotherapy on vascular permeability to take place. Interestingly, our analysis revealed that peri-SRS timing of IT is an independent prognostic factor for OS (from BM diagnosis: HR 0.132, 95% CI 0.034 - 0.517, *P* = 0.004; from SRS: HR 0.14, 95% CI 0.044 - 0.450, *P* = 0.001). Use of IT after SRS led to better OS than concurrent or before (from BM diagnosis: 26.5 vs. 14.1 vs. 7.1 months; from SRS: 21.4 vs. 9.9 vs. 4.1 months, respectively). Several studies have echoed partially similar results regarding IT timing in relation to SRS. Wegner and partners found improved OS in those in whom IT was started at least 21 days after radiotherapy in stage IV NSCLC patients than in those receiving IT within 21 days after radiotherapy (19 vs. 14 months) (43). Schapira and colleagues recorded a longer OS in the recipient of IT post-SRS compared to those who received IT concurrently or before SRS (OS at 1 year, 87.3% vs. 70.0% vs. 0%) (25). A phase 1 KEYNOTE-001 trial revealed that exposure to radiation therapy prior to IT led to better OS than no prior radiation therapy (10.7 vs. 5.3 months) (44). However, some studies claim better OS for both concurrent or IT after SRS. A recent study in the USA reported that adding IT after/concurrent relative to SRS had improved OS than before (13 vs. 3.3 months) (45). Kamran et al. and Cohen-Inbar et al. revealed trends for better OS in the group who received SRS before or during IT administration (26, 46). Contrarily, some insisted that IT and SRS concurrent use is associated with a good OS than before or after (36, 47).

Of note, there seems to be a reasonable agreement that the use of IT prior to SRS is associated with the worst OS compared with concurrent or post-SRS. However, results are inconclusive regarding the concurrent or post-SRS use of IT, which might be due to an inconsistent approach of allocating patients to treatment groups, i.e., some studies considered receipt of IT within 3 months before or after SRS as concurrent, some within 1 month, and some within 2 weeks. In addition, most studies compared concurrent vs. nonconcurrent, prior/during vs. post, and before vs. during/after but rarely before, concurrent, or after, which led to inconclusive results. Although, per current and previous studies trends, there appears to be an optimal time window for IT administration just before SRS or after SRS. Therefore, a larger multi-institutional randomized prospective clinical trial with a more practical approach towards IT timing (with 4 study arms: IT onset immediate before SRS [within 3 weeks prior to SRS], post-SRS early onset of IT, delayed onset, and late-onset) is warranted to identify the optimal time window.

Given the growing use of combined SRS and IT in treating NSCLCBM, traditionally used prognostic tools may not be equally efficient in predicting survival in every BM patient, predominantly in wild-type, since our analysis showed that the median OS has doubled for those who received IT peri-SRS regardless of PD-L1 expression. At present, RPA and DS-GPA are the most widely used prognostic indexes. The RPA index was established based on three consecutive Radiation Therapy Oncology Group (RTOG) trials analysis conducted between 1979 and 1993 (16). RPA introduced three prognostic classes using four clinical variables: age, primary tumor control, KPS, and extra-cranial metastases. Likewise, the GPA index was established in 2008 based on five randomized trials (48). Nevertheless, the classical GPA and RPA indices were all established before the era of IT.

**Figure 5:**
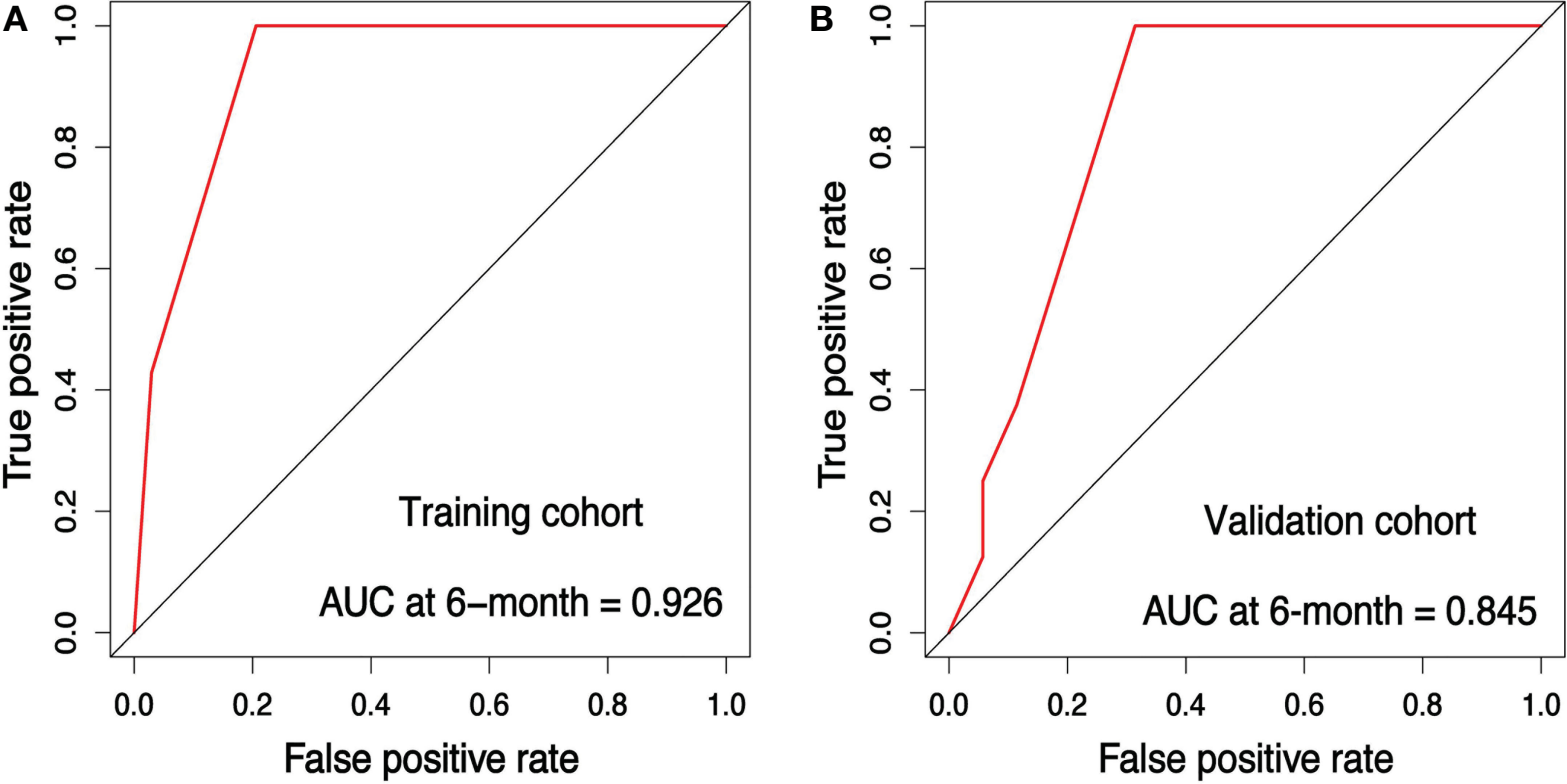
Receiver operating characteristic (ROC) analysis for the training and validation cohort. ROC curve for the prediction model **(A)** in the training cohort **(B)** in the validation cohort.

We comprehensively assessed the prognostic significance of clinicodemographic variables and brain metastatic lesions’ physical characteristics in the present analysis. The latter included the number of BMs, BMs’ distribution, BMs’ location, and CITV (**Table 5**). Multifactor analysis disclosed that IT, RPA, and CITV were the only significant prognostic factors for OS in patients with NSCLCBM. Previously, numerous studies have reported the favorable prognostic role of IT in NSCLCBM patients, which is consistent with our findings (35–37). Likewise, several studies previously reported cumulative tumor volume as an important prognostic factor (19–21). Although the number of BMs is widely considered to impact the patient’s long-term survival, it was not the case in our analysis. Based on multivariate analysis, we developed a nomogram incorporating IT, CITV, and RPA, demonstrating excellent performance. It was found to have a high AUC for the prediction of OS (**Figure 5**).

The internal validation cohort was used to validate the training cohort-based nomogram. Each patient’s risk scores based on the training set’s results were regarded as a variable to conduct Cox proportional hazard regression. The concordance index (C-index) was calculated with the Hmisc package in R to assess the model’s discrimination for prognosis. A C-index of 0.5 designates the absence of discrimination; contrarily, 1.0 indicates a perfect separation of patients with different outcomes. The C-index of a nomogram to predict OS in the training, validation, and overall cohorts were 0.732, 0.665, and 0.690, respectively. The sensitivity and specificity of the nomogram were evaluated by the area under the curve (AUC) of the receiver operating characteristic (ROC) curve using the timeROC package in R. The AUC for the prediction of 6-month OS was 0.926 for the training cohort (**Figure 5A**), 0.845 for the validation cohort (**Figure 5B**), and 0.891 for the overall cohort (**Figure 7A**).

**Figure 6:**
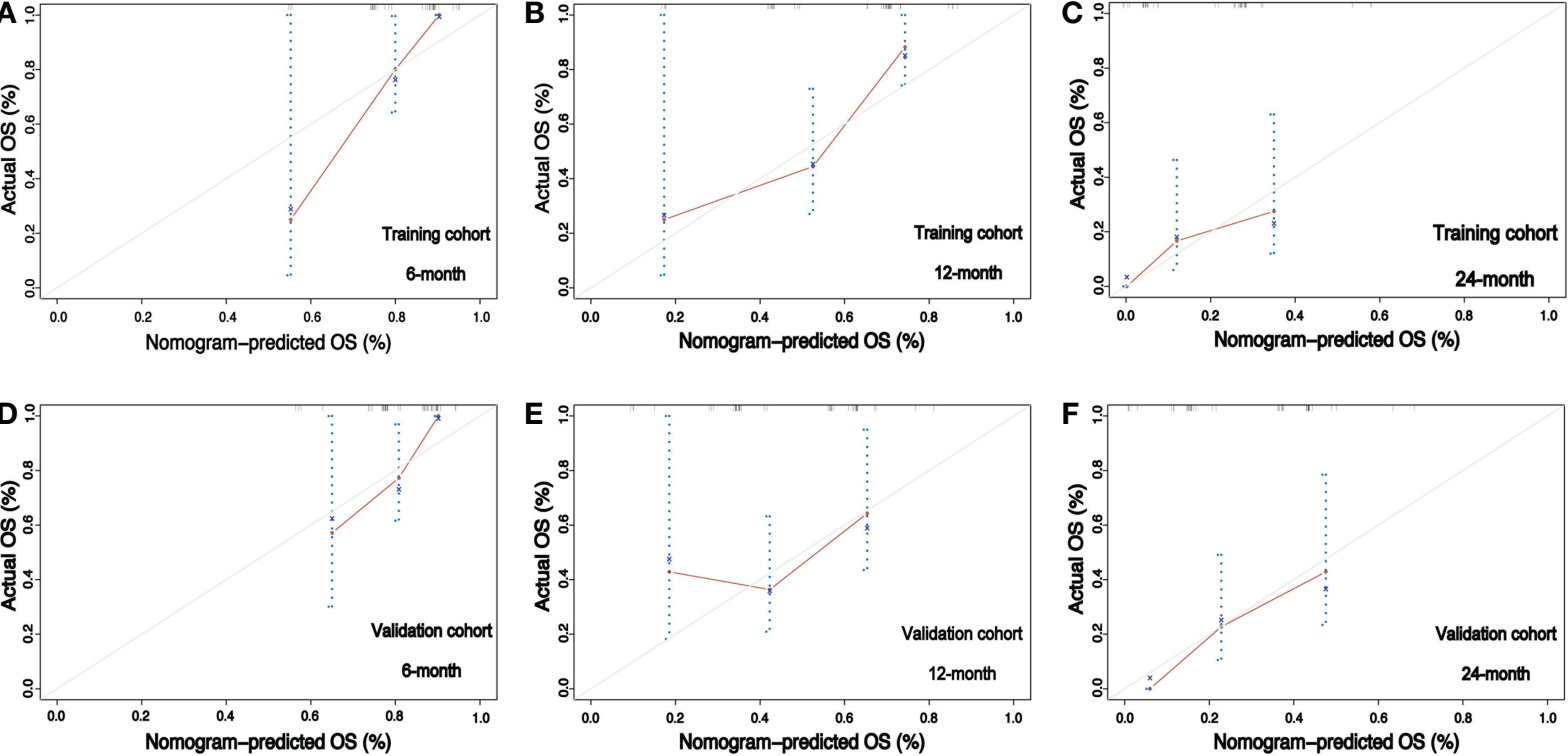
Calibration curves for the training and validation cohorts. **(A–C)** Calibration plots comparing nomogram-predicted and observed overall survival in the training cohort at 6, 12, and 24 months, respectively. **(D–F)** Calibration plots comparing nomogram-predicted and observed overall survival in the validation cohort at 6, 12, and 24 months, respectively.

**Figure 7:**
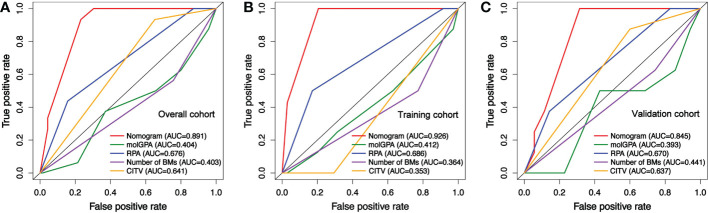
Receiver operating characteristic curves (ROC) comparing the predictive value of the nomogram with lung-molGPA, RPA models, cumulative intracranial tumor volume (CITV), and number of BMs for the prognosis of NSCLCBM patients. **(A)** In the overall cohort, **(B)** in training, and **(C)** in validation cohorts.

Furthermore, the calibration curves plotted through the bootstrap method with 1000 resampling were utilized to reflect the constancy between the actual and nomogram-predicted probability. The calibration plots presented a considerable agreement for the 6-, 12- and 24-month OS in the training and validation sets between the nomogram-predicted and actual OS rates (**Figures 6A-F**). The prediction value of this nomogram was compared with traditionally practiced prognostic indexes, including the lung-molGPA and RPA, as well as the CITV alone and the number of BMs alone. The nomogram established in the present study demonstrated superior discrimination efficacy in training, validation, and overall cohort (**Figures 7A-C**).

**Figure 8:**
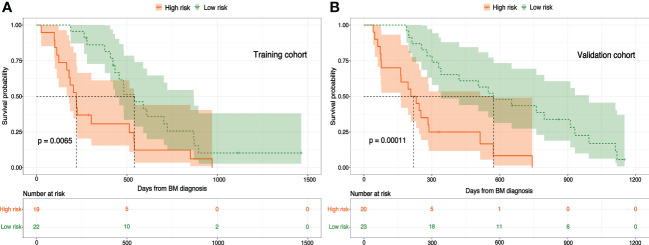
Nomogram-based risk stratifications for NSCLCBM patients and Kaplan–Meier OS curves for patients with low- and high-risk scores in the **(A)** training cohort and **(B)** validation cohort.

According to nomogram predicted risk scores, patients from the training and validation cohort were classified into high-risk and low-risk groups. The Kaplan–Meier survival curve demonstrated a significant difference in OS between low-and high-risk patients in both training and validation cohorts (**Figures 8A, B**). Although the RPA index alone has shown considerable prognosis predictability, its performance was significantly enhanced with the combination of peri-SRS IT, RPA, and CITV. To our knowledge, we are among the first to propose integrating IT and CITV into the RPA index for predicting survival in NSCLCBM patients treated with SRS, especially in those with negative for actionable molecular biomarkers.

Our study had some limitations. The present study is a single-center retrospective cohort with a relatively small sample size. Due to follow-up data limitations, we didn’t estimate the overall response rate (ORR), local control (LC), or progression-free survival (PFS). However, we believe it shouldn’t affect our study outcome as OS is the gold standard endpoint to estimate treatment efficacy. Moreover, several studies previously reported pseudoprogression and abscopal effect in patients who received combined IT and SRS, which makes ORR, LC, and PFS less significant. Over and above, in the real-world setting, patients may undergo multiple rounds of SRS and IT, which can be problematic in dividing patients into groups and may introduce selection bias. Our results might be confounded by other treatment modalities introduced at any time during cancer care. Furthermore, our nomogram was developed based on the training cohort, which was validated with an internal validation cohort but not with an external validation cohort. Further multi-center-based external validation cohort is needed.

Despite these shortcomings inherent to a retrospective study, prolonged OS was evident in patients treated with combined SRS and IT. Receipt of IT post-SRS was associated with better median OS than those who received IT concurrently or before SRS. The adverse events of combined IT and SRS were manageable. Incorporating IT and CITV into the RPA index could augment its survival prediction value in NSCLCBM patients treated with SRS, predominantly in wild-type.

## Data availability statement

The raw data supporting the conclusions of this article will be made available by the authors, without undue reservation.

## Ethics statement

The studies involving human participants were reviewed and approved by Guangdong Sanjiu Brain Hospital ethical committee. Written informed consent for participation was not required for this study in accordance with the national legislation and the institutional requirements.

## Author contributions

Conceptualization: SB, MX, and LC. Methodology: SB, LW, MX, LC, WH, JZ, and ML. Formal analysis and investigation: SB, LW, PZ, MY, YL, HW, YY, XC, RL, GJ, and YG. Writing-original draft preparation: SB. Writing-review and editing: SB, LW, MX, and LC. Supervision: MX and LC. All authors contributed to the article and approved the submitted version.
